# Propagation of societal gender inequality by internet search algorithms

**DOI:** 10.1073/pnas.2204529119

**Published:** 2022-07-12

**Authors:** Madalina Vlasceanu, David M. Amodio

**Affiliations:** ^a^Department of Psychology, New York University, New York, NY 10003;; ^b^Department of Psychology, University of Amsterdam, 1001 NK Amsterdam, The Netherlands

**Keywords:** inequality, algorithm, AI, bias, gender

## Abstract

Humans increasingly rely on artificial intelligence (AI) for efficient and objective decision-making, yet there is increasing concern that algorithms used by modern AI systems produce discriminatory outputs, presumably because they are trained on data in which societal biases are embedded. As a consequence, their use by human decision makers may result in the propagation, rather than reduction, of existing disparities. To assess this hypothesis empirically, we tested the relation between societal gender inequality and algorithmic search output and then examined the effect of this output on human decision-making. First, in two multinational samples (*n* = 37, 52 countries), we found that greater nation-level gender inequality was associated with more male-dominated Google image search results for the gender-neutral keyword “person” (in a nation’s dominant language), revealing a link between societal-level disparities and algorithmic output. Next, in a series of experiments with human participants (*n* = 395), we demonstrated that the gender disparity associated with high- vs. low-inequality algorithmic outputs guided the formation of gender-biased prototypes and influenced hiring decisions in novel scenarios. These findings support the hypothesis that societal-level gender inequality is recapitulated in internet search algorithms, which in turn can influence human decision makers to act in ways that reinforce these disparities.

## Significance

People often rely on artificial intelligence (AI) algorithms to increase their decision-making efficiency and objectivity, yet systemic social biases have been detected in these algorithms’ outputs. We demonstrate that gender bias in a widely used internet search algorithm reflects the degree of gender inequality existing within a society. We then find that exposure to the gender bias patterns in algorithmic outputs leads people to think and act in ways that reinforce societal inequality, suggesting a cycle of bias propagation between society, AI, and users. These findings call for an integrative model of ethical AI that includes human psychological processes to illuminate the formation, operation, and mitigation of algorithmic bias.

Artificial intelligence (AI) algorithms have become pervasive in human decision-making. In virtually all sectors of society, from national security (1) and criminal justice (2) to medicine (3) and education (4), AI algorithms are used to improve the efficiency and accuracy of decisions. One preeminent application is to internet search engines, such as Google Search, which are used daily by people of all ages to gather information that informs their beliefs and behaviors (5). The transfer of such decision power from bias-prone humans to ostensibly objective algorithms has been celebrated as a step toward more fair and rational decision-making (6) and more intelligent problem solving (7).

Yet, despite the promise of objectivity by AI-based decision-making, there is mounting concern about systemic social biases in algorithmic output (8–12). Patterns of discrimination toward women and racial minorities have been detected in algorithms used for hiring decisions (13), delivery of job and housing advertisements (14, 15), university admissions decisions (4), criminal sentencing (2, 16), and health-care allocation (3), among others. For example, Facebook’s job advertisement algorithm targeting users based on their gender was found to disproportionately suggest stereotypically feminine jobs (e.g., nurse or secretary) to women and stereotypically masculine jobs (e.g., janitor or taxi driver) to men, thus further propagating existing gender disparities in the labor market (14).

What causes such biases? A common assumption is that they reflect existing disparities in society represented in the data on which algorithms are trained (17), as well as other aspects of their development, from data collection to data preparation, model development, model evaluation, model postprocessing, and model deployment (18). To the extent algorithms, such as those used widely in internet search engines, encode preexisting social biases (19–21), there is a risk that their outputs could propagate these existing biases (22), thereby reinforcing and strengthening societal and economic inequality. This perspective suggests that AI decision tools could potentially contribute to a cycle of perpetuating inequality, whereby existing social biases are recapitulated in algorithmic outputs, which in turn guide human decisions that reinforce the existing inequality.

To begin to investigate this proposed cycle of bias propagation empirically, we asked whether the degree of inequality within a society relates to patterns of bias in algorithmic output and, if so, whether exposure to such output could influence human decision makers to act in accordance with these biases, thereby reinforcing them. To this end, we first assessed the relationship between existing societal inequality and algorithmic internet search output, in the context of gender, in two multinational studies. Then, we experimentally manipulated exposure to search outputs matching those observed in high- and low-gender inequality nations to test whether they produce corresponding gender bias in human participants’ cognitive concepts and decisions. We hypothesized that the algorithmic outputs of search engines track preexisting societal-level gender biases (H1) and that exposure to these biased algorithmic outputs shape users’ cognitive concepts (H2) and decisions (H3) in manners consistent with societal gender inequality.

In Study 1, we tested the hypothesis that the degree of gender inequality within a society would predict a pattern of gender disparity in internet search output. To do so, we first obtained an open-source dataset known as the Global Gender Gap Index [GGGI (23)], containing rankings of gender inequality for 153 countries. The GGGI represents the magnitude of gender inequality in economic participation and opportunity, educational attainment, health and survival, and political empowerment for each of 153 nations, thus providing societal-level gender inequality scores for each country. Next, to develop an assessment of gender bias in algorithmic output, we drew upon classic research on the prototype bias in cognitive psychology (24, 25), whereby words that should refer with equal probability to a man or a woman, such as “person” (26), or “human” (27), are more often assumed to be a man (28). In recent research using word embeddings trained on massive internet text corpora, words representing the concept of “people” (e.g., “somebody” or “human”) were more likely to cooccur with terms for “men” than for “women”—a demonstration of the male-default bias collectively displayed across individuals in a society (29).

This pervasive gender bias, documented for decades in human cognition, has also been observed in algorithmic outputs. For instance, online translation algorithms make gender-biased errors, assigning a masculine gender to professions such as “doctor” despite the original sentence implying a woman (30). Similarly, image search results for common professions such as “engineer” or “author” underrepresent the true proportion of women in those occupations (31, 32).

On the basis of such biases found in image search results, we developed an approach which involved conducting Google image searches for the keyword “person” within a nation (in its dominant local language) via VPN (virtual private networks; see *Methods* for additional details on this procedure). We chose the term “person” because of its single form even in gendered languages such as Spanish, in which the word for person is “*la persona*” regardless of the gender of the person. In contrast, gendered languages use distinct forms when referring to professionals; in Spanish, for instance, a male doctor is “*el doctor*,” whereas a female doctor is “*la doctora*.” Moreover, in addition to overcoming the constraint posed by gendered languages, the term “person” refers to an aspect of humanity—one’s personhood—that is often undermined as a result of gender discrimination (33). We chose to conduct our search on Google given the overwhelming dominance of its search engine market share worldwide [i.e., over 92% (34)].

We collected data from this search in as many countries as possible. Given nation-specific restrictions and intermittent VPN access to local servers, at the time of Study 1 data collection, VPN connection was successful in 37 countries. A gender bias score was computed for each nation by coding the ratio of man to woman-presenting images within the first 100 results.

**Fig. 1.**
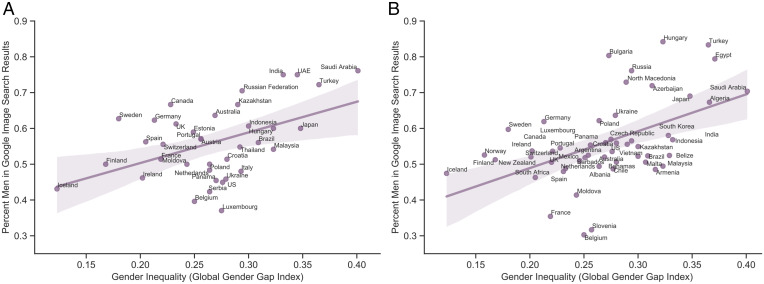
Study 1 (*A*) and Study 2 (*B*) results. Gender inequality (derived from the GGGI computed by the World Economic Forum in 2020; *x* axis) as a function of gender bias in Google image search results, displayed here as the percentage of men in the results of Google images when searching for the gender-neutral keyword "person" in the language and with the IP address of each nation (*y* axis).

To test our central hypothesis, we assessed the relation between Global Gender Gap scores and the degree of gender disparity in image search algorithmic output. This effect was significant, β = 0.472, SE = 0.149, *t* (1,35) = 3.167, CI[0.169, 0.774], *P* = 0.003, such that the proportion of male images was higher in nations with greater gender inequality (computed as the inverse of the GGGI; Fig. 1*A*). This effect remained significant when adjusting for the percentage of women in the population of each nation, β = 0.40, SE = 0.16, *t* (2,34) = 2.47, CI[0.071, 0.729], *P* = 0.019 (*SI Appendix* for additional robustness checks). Thus, in a sample of 37 nations, we found that algorithmic gender bias tracks with societal gender inequality.

We replicated this procedure in Study 2, 3 mo after conducting Study 1, this time obtaining access to internet searches in 52 nations. Replicating Study 1 results, societal gender inequality related to the degree of gender disparity in image search algorithm output, β = 0.50, SE = 0.122, *t* (1,50) = 4.086, CI[0.25, 0.74], *P* < 0.001 (Fig. 1*B*), a pattern that remained significant when adjusting for the percentage of women in the population of each nation, β = 0.49, SE = 0.11, *t* (2,49) = 4.124, CI[0.25, 0.73], *P* < 0.001 (*SI Appendix* for additional robustness checks). Although the man/woman proportions in search output for nations represented in both studies (*n* = 30) differed slightly between the two data collection timepoints as search outputs naturally fluctuate over time (35), the proportions at the two timepoints were highly correlated (*r* = 0.60, *P* = 0.002). Together, Studies 1 and 2 provide empirical evidence consistent with the proposal that societal-level gender disparities are reflected in algorithmic output.

Having demonstrated an association between societal gender inequality and algorithmic search output, we turned to our next question: whether exposure to such algorithmic outputs can shape people’s cognitive concepts and decisions in manners consistent with preexisting societal inequality. Hence, in Study 3, we experimentally tested how exposure to gender ratios in image search results observed for low- versus high-inequality nations in Studies 1 and 2 affect human participants’ judgment and decisions regarding novel categories.

To develop our approach, we built on seminal findings in the social categorization literature, which suggest that people naturally develop category prototypes as a way of assessing and predicting category membership (36). We focused on how such search results would influence judgments and decisions regarding novel categories, given preexisting associations between gender and the concept of personhood. Because novel categories are by definition devoid of such associations, they are optimal for experimentally isolating mechanisms of interest. Here, such mechanisms of interest are the formation of category prototypes—specifically, gender prototypes—and the impact of these prototypes on subsequent consequential decision-making. Thus, the goal of the following experimental investigation was to exemplify the effects of assigning men as the default members, or prototypes, of a category. We chose a labor-market scenario, given the contributing effects of economic participation and opportunity to global gender inequality (23) and in light of findings that algorithmic search results can shape occupational perceptions (31, 32).

**Fig. 2.**
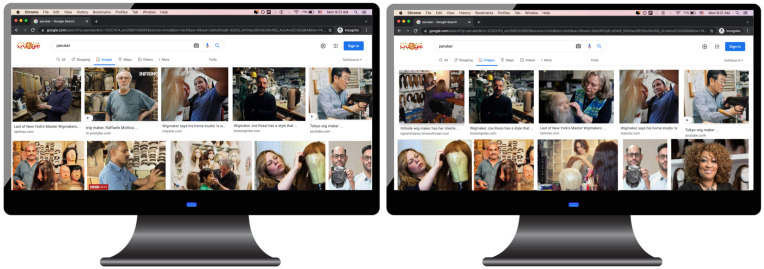
Study 3 stimuli example. Google image search results for the search term “peruker” in the high-inequality condition (90% men, 10% women; *Left*) and the low-inequality condition (50% men, 50% women; *Right*).

In Study 3, 130 participants recruited in the United States (age: mean [M] = 42.1 y, SD = 15.9; 60% women, 39% men, <1% nonbinary) were presented with images depicting the Google image search results of four professions (chandler, draper, peruker, and lapidary). Because these professions are unfamiliar and thus novel to most Americans, our participants would have little prior knowledge about their gender distribution. The gender composition of each profession’s image set was selected to represent the Google image search results for the keyword “person” for nations with the high gender inequality scores (approximately 90% men to 10% women in Hungary or Turkey) and those with low gender inequality scores (approximately 50% men to 50% women in Iceland or Finland) from Study 2 (Fig. 2). This resulted in conditions displaying a high ratio of men to women (9:1, high-inequality) or an equal ratio of men to women of (1:1, low-inequality) performing the profession of interest. Each profession type was randomly assigned to appear in the high-inequality or low-inequality condition, within subjects.

**Fig. 3.**
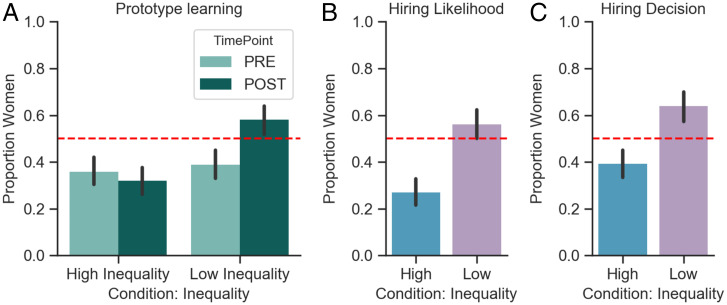
Study 3 results. (*A*) Frequency of “Women” seen as prototypical of the novel category in the high- versus low-inequality conditions, at baseline (light green) and postmanipulation (dark green). (*B*) Proportion of women likely to be hired (postmanipulation) in the high-inequality (blue) condition (χ^2^ = 55.3, w = 0.08, *P* < 0.001) and low-inequality (red) condition (χ^2^ = 3.94, w = 0.02, *P* = 0.047). (*C*) Proportion of women hired (postmanipulation) in the high-inequality (blue) condition (χ^2^= 12.06, w = 0.03, *P* < 0.001) and low-inequality (red) condition (χ^2^ = 19.9, w = 0.05, *P* < 0.001). The red dash lines at *y* = 0.5 represent the chance level gender choice, and the χ^2^ tests reported here compare the distributions to the 0.5 benchmark. Error bars represent 95% CI.

First, prior to viewing image search results, participants made baseline prototypicality judgments regarding each profession (e.g., “Who is more likely to be a peruker, a man or a woman?”). Replicating the classic gender prototype effect (37), participants in both conditions initially judged members of these professions as more likely to be a man (high-inequality: χ^2^ = 21.06, w = 0.05, *P* < 0.001; low-inequality: χ^2^ = 12.94, w = 0.04, *P* < 0.001; Fig. 3*A*), and this effect did not vary by participant gender (*SI Appendix*). Hence, prototypicality judgments did not differ by condition at baseline.

Next, following exposure to image search results, participants again rated the likelihood that a member of each profession was a man or woman. To test our central hypothesis that exposure to images representing the search results for “person” in high vs. low inequality nations influences gender prototypes, these gender prototypicality ratings were submitted to a generalized linear mixed effects model with condition (low vs. high inequality) and time (baseline vs. postmanipulation) as fixed effects and with by-participant and by-item (profession) random intercepts. Our hypothesis was supported by a significant condition by time interaction, β = 0.23, SE = 0.05, *t* = 3.98, Wald Z-test *P* < 0.001 (Fig. 3*A*): Exposure to search result patterns found in low-inequality nations reversed participants’ male-biased prototypes relative to baseline, β = 0.19, SE = 0.04, *t* = 4.76, Wald Z-test *P* < 0.001, whereas exposure to search result patterns found in high-inequality nations appeared to reinforce and thus maintain this prototype, β = 0.03, SE = 0.03, *t* = 0.97, Wald Z-test *P* = 0.32 (Fig. 3*A*). Indeed, the postmanipulation prototype ratings in the low-inequality condition (mean = 0.58, SD = 0.49) were significantly more egalitarian (i.e., closer to the 0.5 benchmark) than those in the high-inequality condition (mean = 0.31, SD = 0.46) in a nonparametric statistical test (i.e., bootstrap, *P* = 0.02). The effect of condition on gender prototypicality judgment did not interact with participants’ gender (*P* = 0.29).

**Fig. 4.**
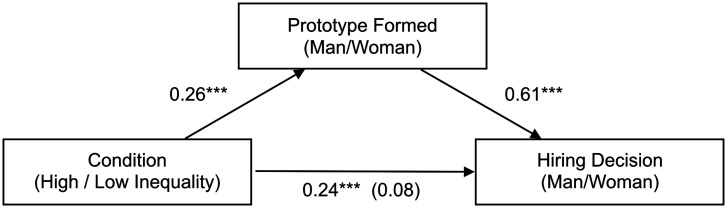
Regression coefficients for the relationship between condition (high/low gender inequality in algorithmic search output) and hiring decision as mediated by the prototype formed. The standardized regression coefficient between condition and hiring decisions, controlling for the prototype formed, is in parentheses.

Following prototype ratings, participants were asked to judge the likelihood that a man or woman would be hired in each profession (“What type of person is most likely to be hired as a peruker?) and then, when presented with images of two job candidates (always one woman and one man) for a position in that profession, to make their own hiring choice (“Choose one of these applicants for a job as a peruker”). As expected, exposure to image sets in the low-inequality condition produced more egalitarian judgments of male vs. female hiring tendencies within a profession, β = 0.292, SE = 0.04, *t* = 7.25, Wald Z-test *P* < 0.001 (Fig. 3*B*), and a higher likelihood of choosing a woman job candidate, β = 0.24, SE = 0.041, *t* = 5.95, Wald Z-test *P* < 0.001 (Fig. 3*C*), compared with exposure to image sets in the high-inequality condition. In a mediation analysis (38), our overarching prediction—that exposure to high- versus low-inequality search results shape gender prototype formation, which in turn influences gender-biased hiring—was supported by a significant indirect effect (Fig. 4 and Tables 1 and 2).

**Table 1.** Regression analyses for the mediation model

Predictors	*b* (SE)	*t*	*F*	df	*R^2^*	*P*
Model 1						
Condition	0.24 (0.04)	5.78***	33.44	(1, 518)	0.058	<0.001
Model 2						
Condition	0.08 (0.03)	2.42*	181.8	(2, 517)	0.41	0.015
Prototype formed	0.61 (0.03)	17.6***			0.05	<0.001

*b* = regression coefficients; df = degrees of freedom. *P < 0.05; ***P < 0.001.

**Table 2.** Causal mediation analyses: Nonparametric bootstrap CI, with 10,000 simulations

	Estimate	95% CI lower	95% CI upper	*P*
Indirect effect (ACME)	0.161	0.109	0.22	<0.001***
Direct effect (ADE)	0.084	0.015	0.16	0.018*
Total effect	0.246	0.163	0.33	<0.001***
Proportion mediated	0.656	0.470	0.92	<0.001***

ACME = average causal mediation effects; ADE = average direct effect. *P < 0.05; ***P < 0.001.

To further probe the impact of search output exposure on prototype formation and decision-making, we next assessed the effects of biased algorithmic outputs in nations in which search results are biased in favor of women. Study 4 (*n* = 137, mean age = 41.81 y, SD = 15.17; 57.6% women) repeated the design of Study 3 but selected the composition of image sets for each profession to represent nations yielding the highest and lowest male:female ratios in the Study 2 Google image search results for the keyword “person” (approximately 90% men to 10% women in Hungary or Turkey, and approximately 30% men to 70% women in Belgium). Hence, the conditions in this study displayed a higher proportion of either men (9:1, majority-men condition) or women (7:3; majority-women condition) performing the profession of interest. We found that search output exposure biased the prototype formation symmetrically, suggesting a contrast effect: In the majority-men condition, the prototype became more male, β = 0.1058, SE = 0.038, *t* = 2.758, Wald Z-test *P* = 0.005, whereas in the majority-women condition, the prototype became more female, β = 0.3211, SE = 0.037, *t* = 8.466, Wald Z-test *P* < 0.001. This result conceptually replicates canonical prototype formation effects (38, 39) in the context of exposure to algorithmic outputs (*SI Appendix* for additional information).

Study 4 participants also more frequently selected men as more likely to be hired in a majority-men condition and women in a majority-women condition, β = 0.397, SE = 0.038, *t* = 10.40, Wald Z-test *P* < 0.001 (*SI Appendix* for additional information). An additional study (Study 5, *n* = 128; mean age = 39.6 y, SD age = 13.34; 61% women) replicated these results and further showed that exposure to majority-women image results increased decisions to hire women compared with exposure to majority-men search results (β = 0.33, SE = 0.041, *t* = 8.046, Wald Z-test *P* < 0.001; *SI Appendix* for additional information).

## Discussion

Social biases have been widely observed in AI algorithms used for a variety of decision tools, and it has been proposed that such biases are rooted in existing societal disparities (8–12). Here, we provide empirical evidence in support of this proposal: In two cross-national studies of 37 (Study 1) and 52 (Study 2) countries, higher national levels of gender inequality predicted a larger male/female disparity in Google image search results for the gender-neutral search term “person.” That is, societal-level gender inequality was reflected in search algorithm output.

We then documented the effect of these search output disparities on human users’ gender concepts and decisions in a novel labor-market scenario. When exposed to search result patterns from high-inequality nations in this context, participants were more likely to form male prototypes of a profession, expect men to be hired in that profession, and select men for a position in that profession. Exposure to search result patterns from low-inequality nations eliminated this effect: Participants were somewhat more likely to conceptualize, expect, and choose to hire more women. Mediation analyses indicated that gender disparities in search results influenced participant’s gender prototypes, which in turn guided their hiring decisions. Finally, we found that exposure to search results depicting higher proportions of women fully reversed these preferences, with prototypes, expectations, and decisions strongly favoring women over men. Remarkably, a one-shot exposure to the biased search output was enough to generate correspondingly biased judgements and decisions. In everyday usage, however, people are likely exposed to many such algorithmic outputs, which may exert a cumulative impact on users’ cognitive representations and their behavioral signatures.

Together, these findings demonstrate that societal levels of inequality are evident in internet search algorithms and that exposure to this algorithmic output can lead human users to think and potentially act in ways that reinforce this societal inequality. Our studies examined a specific test case of this pattern, in the context of gender inequality in image search algorithms and their experimental effects in novel decision domains; although real-world decisions are often more complex, this approach afforded a clear and valid proof of concept for the proposed mechanism of algorithmic bias propagation. However, algorithms vary widely in their functions, implementation, and usage. The extent to which they represent societal biases and influence users to act in accordance is also subject to variance. Nevertheless, evidence for algorithmic bias is already abundant (8–12), and its influence on human users, even when subtle, is likely to be pervasive (32).

Our findings further illuminate the impact of algorithmic bias on human cognition by demonstrating its effect on prototype formation. Given the widespread reliance on internet search engines in both formal education and daily life (40), people’s cognitive concepts are likely shaped to a large extent by the outputs of computer algorithms (39). Our research experimentally demonstrates that when these outputs are biased, people’s cognitive concepts (e.g., the prototypical person, the prototypical professional) can be shifted accordingly. As shown here and in prior work (41), these shifts can then trigger decisions congruent with a skewed reality that discriminate against those who do not fit one’s conception of a social category’s typical members. Moreover, when the mere concept of personhood overlaps mainly with one gender, the objectification of nonprototypical individuals can lead to additional discrimination (33) and oppression (42). Given the nation-level disparities observed in Studies 1 and 2, the effects of search algorithms on human users’ concepts and worldviews may be disproportionately severe in societies with greater existing gender inequality.

It is notable that this research was conducted using a VPN-based internet search methodology. This approach allowed us to probe international differences in internet search algorithm function with low financial and administrative costs. However, national VPN access varies, such that it may be banned, obstructed, or otherwise unavailable in many locations (43) and thus may not provide an exhaustive international assessment. Nevertheless, this method provided us with access to 58 nations spanning six continents, with sufficient variability in national gender inequality to permit a valid test of our hypothesis. Thus, while VPN-based research may be limited to nonexhaustive cross-national assessments, it offers an effective tool for testing societal-level effects of internet-based algorithms.

Finally, by demonstrating a link between societal inequality, internet search algorithms, and human decision-making, our findings highlight potential intervention points for bias reduction efforts. Whereas past research has focused on interventions aimed at debiasing an algorithm’s training set (44, 45) and elucidating the computations of deep neural network models (46), our findings suggest that to increase transparency and fairness in AI it will also be critical to uncover how human decision makers interact with and consume algorithmic output (47, 48). Psychology-based interventions aimed at breaking the cycle of bias propagation between society and AI might be particularly impactful. More broadly, our findings call for an integrative model of ethical AI that combines human psychology with computational and sociological approaches to illuminate the formation, operation, and mitigation of algorithmic bias.

## Methods

### Methods Study 1.

#### Open science practices.

Data can be found on the study’s Open Science Framework (OSF) page. The data-analysis code (in Python) can be accessed as a Jupyter notebook on GitHub.

#### Measures.

To compute a measure of algorithmic gender bias, a research assistant, blind to the study’s hypotheses, coded the ratio of women- to men-presenting images of the first 100 results in Google image search using the gender-neutral keyword “person,” translated (with Google Translate and using back-translation verification) in the language of each of the nations in which the search was conducted (Table 3). Of note, although some of these languages have gendered nouns (e.g., Spanish and Arabic) and others do not (e.g., English and Turkish), the gender proportion of search outputs obtained in gendered and nongendered languages did not differ in either Study 1 (*P* = 0.093) or Study 2 (*P* = 0.597). Moreover, the research assistant was instructed to conduct the search in all the 153 countries included in the 2020 Global Gender Gap Report (23). To conduct the search from different locations around the world, we used a VPN service (https://www.le-vpn.com) that activates Internet Protocol (IP) addresses corresponding to nations of interest. For each location activated, we used Google’s IP address identifier to ensure the IP address under which each search was conducted was the intended one. In between each search, the research assistant cleared the browser history of the computers used to collect and code the data, in order to avoid contamination of the search results. Using this method, the VPN connection was successful in 37 nations (Table 3). The dataset was collected in August 2021.

**Table 3.**
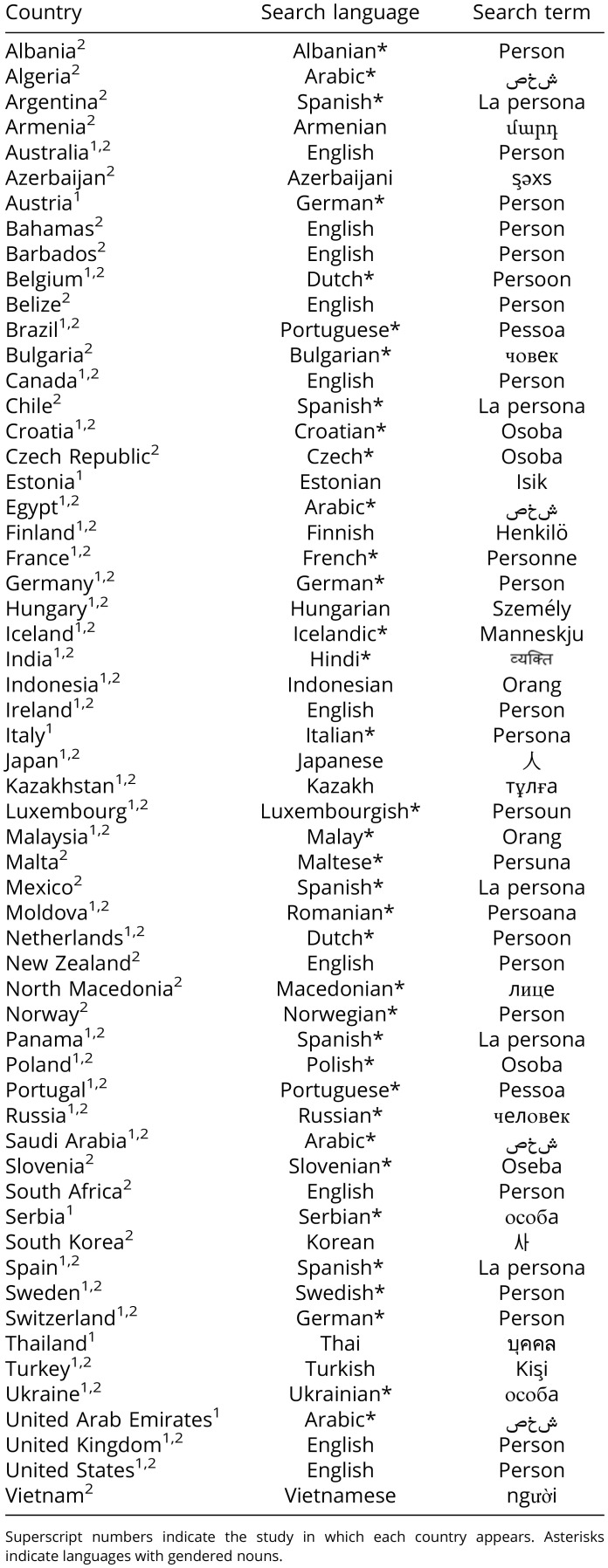
Language used to collect data from the countries represented in the dataset

The GGGI, published in the 2020 Global Gender Gap Report (23), was used as an index of societal-level gender inequality. The GGGI was introduced by the World Economic Forum in 2006 to capture the magnitude of gender disparity in economic participation and opportunity, educational attainment, health and survival, and political empowerment. According to these criteria, the index ranks each nation and provides them a score ranging from 0 to 1, 1 corresponding to full gender parity. For example, the highest score (0.82) was achieved by Iceland, meaning that this nation has closed 82% of its gender gap. We used this index to compute a measure of societal gender inequality, which we derived as one minus the GGGI index, such that higher values represent higher inequality.

### Methods Study 2.

#### Open science practices.

Data can be found on the study’s OSF page. The data-analysis code (in Python) can be accessed as a Jupyter notebook on GitHub.

**Fig. 5.**
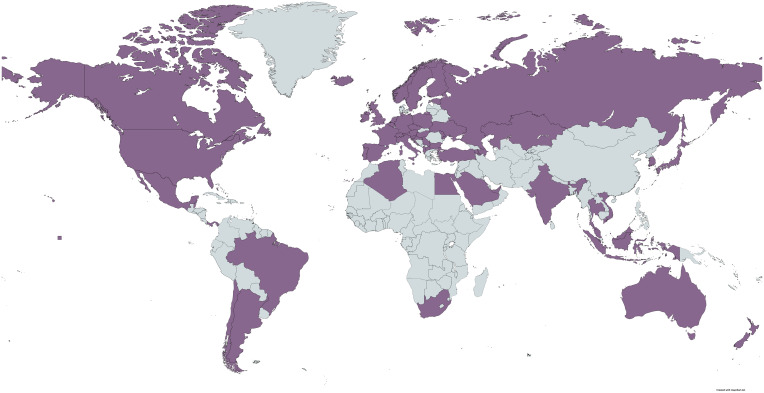
Nations represented in the datasets of Studies 1 or 2 (i.e., in which the VPN connection was successful during at least one of the two data collection timepoints). Map created using https://www.mapchart.net/ (creative commons attribution license).

The same data collection procedure was followed as in Study 1. A different research assistant, also blind to the study’s hypotheses, performed the image scraping and coding procedure. The dataset for Study 2 was collected in November 2021. The VPN connection was successful in 52 nations (Table 3). In total, across Studies 1 and 2, data were successfully collected from 58 nations (Fig. 5).

### Methods Study 3.

#### Open science practices.

The study’s experimental design and hypotheses were preregistered at AsPredicted, and stimuli and data are available on the study’s OSF page. The data-analysis code (in Python) can be accessed as a Jupyter notebook on GitHub.

#### Participants.

We estimated that to obtain a power of 0.80 to detect a moderate effect size (Cohen’s d) of 0.25 in a paired-sample comparison, a sample of 128 participants would be needed. Participants were recruited on Cloud Research (https://www.cloudresearch.com/), an Internet-based research platform similar to Amazon Mechanical Turk but with more intensive participant pool checks. Participants were compensated at the platform’s standard rate (49). In total, we recruited 136 participants, of which 6 were excluded from the analysis on the basis of preregistered criteria (i.e., attention checks). The final sample contained 130 participants (age: M = 42.1 y, SD = 15.9; 60% women, 39% men, <1% nonbinary). The study protocol was approved by the New York University Institutional Review Board.

#### Stimuli materials.

We designed “screenshots” of Google image search results for four professions (i.e., chandler, draper, peruker, and lapidary) of which participants were unlikely to have previous knowledge. For each profession, we designed screenshots of both men-dominated search results (high-inequality condition) and gender-balanced search results (low-inequality condition). In designing the stimuli, we used real images from Google image search, but we manipulated the proportion in which they appeared in the search result page.

#### Design and procedure.

Data were collected in February 2022. Participants were told they would participate in a study about professions and were directed to the Qualtrics platform. After providing informed consent, participants were directed to the pretest phase in which they were asked a series of questions about each of the four professions (e.g., chandler), such as “What is a chandler?,” “What is the age range of a person most likely to work as a chandler?,” and “Who is more likely to be a chandler?” with options being “a man” or “a woman.” Participants were also asked to estimate the salary range of a typical chandler, as well as the degree of friendliness and intelligence of the typical chandler. Then, participants were told they would see the screenshot of the Google image search results for the keyword “chandler,” after which they were randomly assigned to either the high-inequality condition, in which the search results displayed 90% men and 10% women working as a chandler, or the low-inequality condition, in which the search results displayed 50% women and 50% men working as a chandler. These proportions were selected to represent the Study 2 Google image search results for the keyword “person” within nations with high gender inequality (approximately 90% men and 10% women in Hungary or Turkey), and low gender inequality (approximately 50% men and 50% women in Iceland or Finland). After seeing these image search results, participants were directed to the posttest phase, in which they were asked all the pretest questions again. Finally, participants were asked “What type of person is most likely to be hired as a chandler?,” after which they entered a hiring decision task in which they were asked to choose between two applicants (a man and a woman) for each profession they learned about in the study. Participants were shown an image of each applicant and were asked to make a hiring decision in a forced-choice task. Finally, they completed a series of demographic questions and were debriefed.

### Methods Study 4.

#### Open science practices.

The study’s experimental design and hypotheses were preregistered at AsPredicted. In addition, the stimuli and data can be found on the study’s OSF page. The data-analysis code (in Python) can be accessed as a Jupyter notebook on GitHub.

#### Participants.

We estimated that to obtain a power of 0.80 to detect a moderate effect size (Cohen’s d) of 0.25 in a paired sample comparison, a sample of 128 participants would be needed. Participants were recruited on Cloud Research and were compensated at the platform’s standard rate (49). In total, we recruited 139 participants, of which 2 were excluded from the analysis on the basis of preregistered criteria (i.e., attention checks). The final sample contained 137 participants (age: M = 41.81 y, SD = 15.17; 57.6% women, 42% men, <1% nonbinary). The study protocol was approved by the New York University Institutional Review Board.

#### Stimuli materials.

We used the same materials as in Study 3, with one exception: The gender composition of images sets for each profession were now selected to represent the Study 2 Google image search results for the keyword “person” within nations with the highest search output proportion in favor of men (approximately 90% men and 10% women in Hungary or Turkey) and nations with the highest search output proportion in favor of women (approximately 30% men and 70% women in Belgium). This resulted in conditions displaying a higher proportion of either men (9:1) or women (7:3) performing the profession of interest. Each profession was randomly assigned to appear in the majority men or majority women condition, within subjects.

#### Design and procedure.

The data were collected in December 2021. The design and procedure were the same as in Study 3, with one exception: In Study 4 we did not include the hiring decision task.

### Methods Study 5.

#### Open science practices.

The study’s experimental design and hypotheses were preregistered at AsPredicted. In addition, the stimuli and data can be found on the study’s OSF page. The data-analysis code (in Python) can be accessed as a Jupyter notebook on GitHub.

#### Participants.

We estimated that to obtain a power of 0.80 to detect a moderate effect size (Cohen’s d) of 0.25 in a paired sample comparison, a sample of 128 participants would be needed. Participants were recruited on Cloud Research and were compensated at the platform’s standard rate (49). In total, we recruited 131 participants, of which 3 were excluded from the analysis on the basis of preregistered criteria (i.e., attention checks). The final sample contained 128 participants (age: M = 39.6 y, SD = 13.34; 61% women, 38% men, <1% nonbinary). The study protocol was approved by the New York University Institutional Review Board.

#### Stimuli materials.

We used the same materials as in Study 4.

#### Design and procedure.

The data were collected in January 2022. The design and procedure were the same as in Study 4, with one exception: In Study 5 we added a hiring decision task at the end of the study, in which participants were asked to choose between two applicants (a man and a woman) for the profession in question. Participants were shown an image of each applicant and were asked to make a hiring decision in a forced choice task.

## Supplementary Material

Supplementary File

## Data Availability

Study data, materials, and code have been deposited in OSF (https://osf.io/scvjk/) (50) and GitHub (https://github.com/mvlasceanu/AIbias) (51).
