# Supplementary material for: Propagation of societal gender inequality by internet search algorithms
Source: Proc Natl Acad Sci U S A. 2022 Jul 12;119(29):e2204529119. doi: 10.1073/pnas.2204529119 (PMC9304000; doi:10.1073/pnas.2204529119)
Supplement: Supplementary File [file pnas.2204529119.sapp.pdf]

Supplementary Information

Study 1 Results

In studies using nation-level indices, such as the Global Gender Gap Index (GGGI), a potential concern is that scores for adjacent nations may not be completely independent due to overlap in culture, language, or societal structures. Although our broad hypothesis—that societal gender inequality would be evident in internet search output—could apply to nations as well as regions, we focused on a national level of analysis in part because internet search algorithms operate within national firewalls.

Given the concern about nonindependence in nation-level indices like the GGGI, we assessed whether an association between national gender inequality score and algorithmic output would emerge even among nations within a global region. To do so, we tested a hierarchical model nesting the nations into world regions (Table S1), to control for the nations in our dataset potentially providing non-independent datapoints in terms of the Global Gender Gap Index, following procedures by Kuppens and Pollet (2015). Despite this being a conservative test, given that it removed variance of interest linked to regions and was relatively underpowered, due to some regions including as few as a single nation, the effect remained significant. That is, when nesting data by world region in a linear mixed effect model with gender disparity in image search algorithmic output as the dependent variable, the GGGI as a fixed effect, including by region random intercepts, national gender inequality significantly predicted the gender disparity in image search results (β =0.46, SE=0.15, $t(8)=2.962$, $p=0.016$). This result suggests that our finding is robust to the concern of nonindependence in nation-level indices.

Study 2 Results

As in Study 1, we conducted a robustness check of the main finding by running a hierarchical model nesting the countries into world regions (Table S1). The effect of national gender inequality on gender disparity in image search results remained significant in a linear mixed effect model with gender disparity in image search algorithmic output as the dependent variable, the GGGI as a fixed effect, and including by region random intercepts (β =0.50, SE=0.12, $t(50)=4.08$, $p<0.001$). Again, this is a conservative test because it removes meaningful variance represented at the regional level. Nevertheless, is demonstrates that our findings are robust to potential concerns of nonindependence of nation-level scores.

Table S1. World region used to categorize the countries represented in the dataset following procedures by Kuppens and Pollet (2015). Superscripts indicate the study in which each country appears.

Country	Region	Country	Region
Albania[2]	Central and Eastern Europe	Kazakhstan[1,2]	Asia
Algeria[2]	Middle East and North Africa	Luxembourg[1,2]	Northern and Western Europe
Argentina[2]	South America	Malaysia[1,2]	South and Southeast Asia
Armenia[2]	Central and Eastern Europe	Malta[2]	Northern and Western Europe
Australia[1,2]	Anglo-Saxon countries	Mexico[2]	Central America and Caribbean
Azerbaijan[2]	Asia	Moldova[1,2]	Central and Eastern Europe
Austria[1]	Northern and Western Europe	Netherlands[1,2]	Northern and Western Europe
Bahamas[2]	Central America and Caribbean	New Zealand[2]	Anglo-Saxon countries
Barbados[2]	Central America and Caribbean	North Macedonia[2]	Central and Eastern Europe
Belgium[1,2]	Northern and Western Europe	Norway[2]	Northern and Western Europe
Belize[2]	Central America and Caribbean	Panama[1,2]	Central America and Caribbean
Brazil[1,2]	South America	Poland[1,2]	Central and Eastern Europe
Bulgaria[2]	Central and Eastern Europe	Portugal[1,2]	Northern and Western Europe
Canada[1,2]	Anglo-Saxon countries	Russia[1,2]	Asia
Chile[2]	South America	Saudi Arabia[1,2]	Middle East and North Africa

Croatia[1,2]	Central and Eastern Europe	Slovenia[2]	Central and Eastern Europe
Czech Republic[2]	Central and Eastern Europe	South Africa[2]	Africa
Estonia[1]	Northern and Western Europe	Serbia[1]	Central and Eastern Europe
Egypt[1,2]	Middle East and North Africa	South Korea[2]	Asia
Finland[1,2]	Northern and Western Europe	Spain[1,2]	Northern and Western Europe
France[1,2]	Northern and Western Europe	Sweden[1,2]	Northern and Western Europe
Germany[1,2]	Northern and Western Europe	Switzerland[1,2]	Northern and Western Europe
Hungary[1,2]	Central and Eastern Europe	Thailand[1]	South and Southeast Asia
Iceland[1,2]	Northern and Western Europe	Turkey[1,2]	Central and Eastern Europe
India[1,2]	South and Southeast Asia	Ukraine[1,2]	Central and Eastern Europe
Indonesia[1,2]	South and Southeast Asia	UAE[1]	Middle East and North Africa
Ireland[1,2]	Anglo-Saxon countries	UK[1,2]	Anglo-Saxon countries
Italy[1]	Northern and Western Europe	US[1,2]	Anglo-Saxon countries
Japan[1,2]	Asia	Vietnam[2]	South and Southeast Asia

Study 3 Results

In a generalized linear mixed effects model with baseline gender prototypicality (man/woman) as the dependent variable, condition (low/high inequality) as the fixed effect, and by-participant and by-item random intercepts, we found that the frequency of men inferred as the professions' prototype was not different in the two conditions, β = 0.03, SE = 0.04, t = 0.726, Wald Z-test p-value = 0.467.

Moreover, participants' gender did not affect the baseline prototypicality judgements - in a generalized linear mixed effects model with baseline gender prototypicality (man/woman) as the dependent variable, participant gender (man/woman) as the fixed effect, and by-participant and by-item random intercepts, the frequency of men inferred as the professions' prototype was similar for men and women participants, β = 0.05, SE = 0.04, t = 1.314, Wald Z-test p-value = 0.188. When introducing both condition and participant gender in the model, the condition by participant gender interaction at baseline was not significant (β = 0.6, SE = 0.08, t = 0.769, Wald Z-test p-value = 0.442).

Participants' gender also did not modulate the effect of condition on the change in prototypicality judgements: in a generalized linear mixed effects model with gender prototypicality (man/woman) as the dependent variable, participant gender (man/woman), condition (high/low inequality) and time (baseline vs. post-manipulation) as fixed effects, including by-participant and by-item random intercepts, we found a non-significant three way interaction (β = 0.007, SE = 0.11, t = 0.063, Wald Z-test p-value =0.949). We also tested whether there was an effect of condition on salary range estimates, friendliness estimates, and competence estimates of the "typical" person in each profession about which participants learned,in three different linear mixed effects models with condition (high/low inequality) as the fixed effect, and by-participant and by-item random intercepts. We found no significant effect of condition on salaryestimates (β = 0.64, SE = 1.09, t = 0.589, p-value = 0.556), competence estimates (β = 0.77, SE = 0.93, t = 0.838, p-value = 0.403), or friendliness estimates (β = 0.95, SE = 1.06, t = 0.89, p-value = 0.372).

Study 4 Results

We assessed whether portraying a profession as dominated by men or dominated by women created prototypicality judgements, in generalized linear mixed models (for each condition) with gender prototypicality (man/woman) as the dependent variable, time (baseline vs. post-manipulation) as the fixed effect,and by-participant and by-item random intercepts. Results indicated that the frequency of men inferred as the professions' prototype increased from pre-test to post-test in the majority men condition, β = 0.1058, SE =0.038, t = 2.758, Wald Z-test p-value = 0.005 (Figure S1A), and the frequency of women attributed as theprofessions' prototype increased from pre-test to post-test in the majority women condition, β = 0.3211, SE = 0.037, t = 8.466, Wald Z-test p-value < 0.001 (FigureS1B). Thus, we found

that gender bias in the outputs of search algorithms can influence people's cognitive concepts by facilitating the formation of gender prototypes.

We also assessed the downstream consequences of gender prototype formation from biased algorithmic outputs. Participants in Study 4 were also asked to estimate whether a man or a woman would be more likely to be hired in each profession. In a generalized linear mixed models with hiring likelihood (man/woman) as the dependent variable, condition (majority men/women) as the fixed effect, and by-participant and by-item random intercepts, we found a significant effect of condition, $\beta = 0.397$, SE = 0.038, t = 10.40, Wald Z-test p-value < 0.001 (Figure S1C), suggesting that when the search output displayed a majority of men, participants preferentially selected men as the more likely candidates for the profession in question, showing a bias against women, and vice versa. Therefore, we also found an effectof gender prototype on hiring likelihood estimates.

We also tested whether there was an effect of condition on salary range estimates, friendliness estimates, and competence estimates of the "typical" person in each profession participants learned about, in three different linear mixed effects models with condition (high/low inequality) as the fixed effect, and by-participant and by-item random intercepts. We found no significant effect of condition on salary estimates ($\beta = 0.94$, SE = 1.04, t = 0.908, p-value = 0.364) or competence estimates ($\beta = 0.63$, SE = 1.02, t = 0.618, p-value = 0.53), but a significant effect of condition on friendliness estimates ($\beta = 2.89$, SE = 1.08, t = 2.67, p-value < 0.0078), such that professionals displayed in the low inequality condition were rated as more friendly than professionals displayed in the high inequality condition.

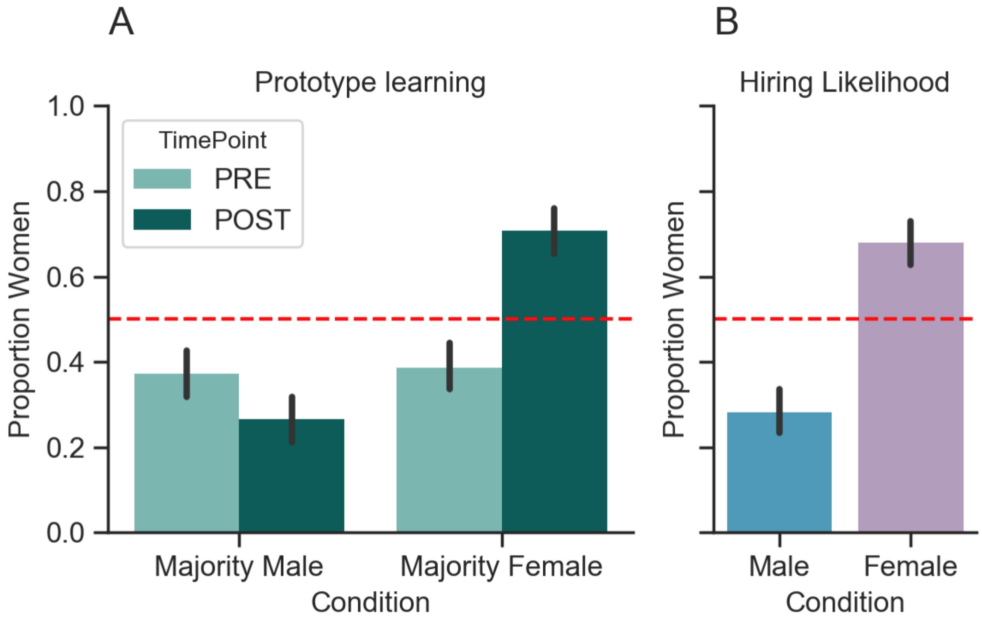

Figure S1. Study 4 Results. Panel A: Frequency of 'Women' seen as prototypical of the novel category in the majority men and women condition, at pre-test (light green) and post-test (dark green). Panel B: Proportion of women likely to be hired in the majority men (blue) versus women (red) conditions.

Study 5 Results

We assessed whether gender prototype formation as a function of image search results can also trigger actual discriminatory hiring decisions in a labor market scenario. We recruited another sample of 128 participants (age: M = 39.6 years, SD = 13.34; 61% women) and replicated the procedure used in Study 4, with one exception: we added a hiring decision task in which participants were asked to decide (a forced

choice task) between 2 applicants (a man and a woman) for each profession.

First, we replicated all the results found in Study 4. We again found that the frequency of men attributed as the prototype in the majority men condition increased from pre-test to post-test $\beta = 0.1132$, SE = 0.037, t = 2.985, p = 0.002 (Figure S2A). Similarly, the frequency of women attributed as the prototype in the majority women condition increased from pre-test to post-test $\beta = 0.4179$, SE = 0.038, t = 10.721, p < 0.001 (Figure S2A). We also replicated the effect of prototype formation on hiring likelihood estimates, $\beta = 0.472$, SE = 0.038, t = 12.20, p < 0.001 (Figure S2B).

To assess the effect of prototype formation as a function of image search results on hiring decisions, we ran a generalized linear mixed model with hiring decision (man/woman) as the dependent variable, condition (man/woman) as the fixed effect, and by-participant and by-item random intercepts, and found a significant effect of condition such that women were more likely to be hired in the woman compared tothe man condition, $\beta = 0.33$, SE = 0.041, t = 8.046, Wald Z-test p-value < 0.001 (Figure S2C).

Finally, we again tested whether there was an effect of condition on salary range estimates, friendliness estimates, and competence estimates of the "typical" person in each profession participants learned about, in three different linear mixed effects models with condition (high/low inequality) as the fixed effect, and by-participant and by-item random intercepts. We found no significant effect of condition on salary estimates ($\beta = 1.95$, SE = 1.31, t = 1.48, p-value = 0.139), competence estimates ($\beta = 0.53$, SE = 1.00, t = 0.534, p-value = 0.593), or friendliness estimates ($\beta = 1.28$, SE = 1.17, t = 1.08, p-value = 0.277).

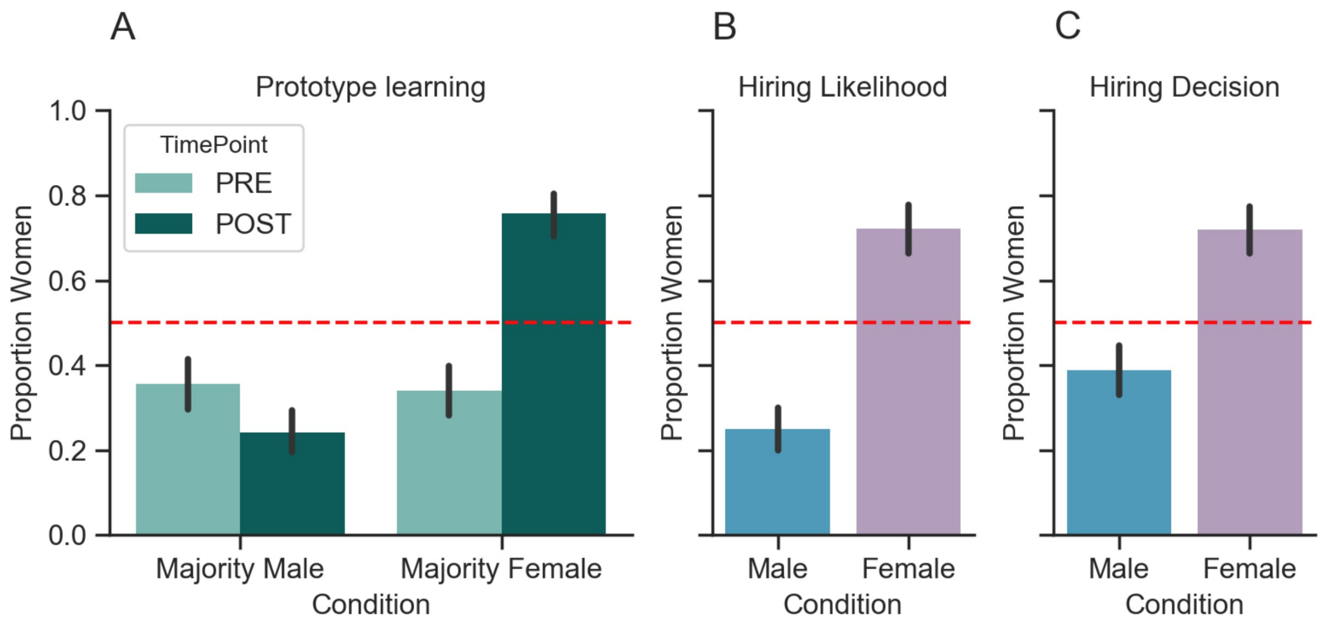

Figure S2. Study 5 Results. Panel A: Frequency of 'Women' seen as prototypical of the novel category in the Man and Woman Conditions, at pre-test (light green) and post-test (dark green). Panel B: Proportion of women likely to be hired in the Man (blue) versus Woman (red) conditions.
